# Single cardiomyocyte nuclear transcriptomes reveal a lincRNA-regulated de-differentiation and cell cycle stress-response in vivo

**DOI:** 10.1038/s41467-017-00319-8

**Published:** 2017-08-09

**Authors:** Kelvin See, Wilson L. W. Tan, Eng How Lim, Zenia Tiang, Li Ting Lee, Peter Y. Q. Li, Tuan D. A. Luu, Matthew Ackers-Johnson, Roger S. Foo

**Affiliations:** 1 Genome Institute of Singapore, 60 Biopolis Street, Singapore, 138672 Singapore; 2 Cardiovascular Research Institute, National University Health System, Centre for Translational Medicine, 14 Medical Drive, Singapore, 117599 Singapore

## Abstract

Cardiac regeneration may revolutionize treatment for heart failure but endogenous progenitor-derived cardiomyocytes in the adult mammalian heart are few and pre-existing adult cardiomyocytes divide only at very low rates. Although candidate genes that control cardiomyocyte cell cycle re-entry have been implicated, expression heterogeneity in the cardiomyocyte stress-response has never been explored. Here, we show by single nuclear RNA-sequencing of cardiomyocytes from both mouse and human failing, and non-failing adult hearts that sub-populations of cardiomyocytes upregulate cell cycle activators and inhibitors consequent to the stress-response in vivo. We characterize these subgroups by weighted gene co-expression network analysis and discover long intergenic non-coding RNAs (lincRNA) as key nodal regulators. KD of nodal lincRNAs affects expression levels of genes related to dedifferentiation and cell cycle, within the same gene regulatory network. Our study reveals that sub-populations of adult cardiomyocytes may have a unique endogenous potential for cardiac regeneration in vivo.

## Abstract (web summary)

Adult mammalian cardiomyocytes are predominantly binucleated and unable to divide. Using single nuclear RNA-sequencing of cardiomyocytes from mouse and human failing and non-failing adult hearts, See et al. show that some cardiomyocytes respond to stress by dedifferentiation and cell cycle re-entry regulated by lncRNAs.

## Introduction

In the lifetime of an adult mouse or human heart, new cardiomyocytes (CMs) are generated albeit at very low rates of ~1%^1–3^. On the other hand, adult zebrafish and neonatal mouse hearts can fully regenerate upon surgical resection or infarct injury^4–6^. Like the zebrafish and neonatal mouse, new CMs in the adult mouse appear to arise by mitosis of pre-existing CMs^1, 2^, but a sufficient level of endogenous mitosis is lacking to allow for adequate regeneration and repair during disease progression^7, 8^. Loss of the full capacity to regenerate occurs soon after the seventh postnatal day (P7) when CMs in the neonatal mouse heart exit the cell cycle^4^.

This highlights two key questions for the field of cardiac regeneration: (a) what holds back adult CMs from dividing and (b) can any adult CM be induced to divide? Indeed lineage tracing studies in regenerating hearts of zebrafish^5^ and neonatal mice^4^, show that proliferation potency is achieved by cell cycle re-entry of pre-existing CMs. Consistent with this, Hippo/Yap pathway components^9, 10^, the transcription factor *Meis1* ^11^, and a series of microRNA including members of the miR-15 family^12^, miR-199a, miR-590^13^, miR-17-92 cluster^14^, miR-99/10, and Let-7a/c^15^ have been separately implicated in the regulation of CM proliferation. Kimura *et al*.^16^, showed that while the majority of CMs in adult mouse hearts permanently exit the cell cycle, a rare subset existing in relatively hypoxic microenvironment of the myocardium, retain proliferative neonatal CM features, and have smaller size, mono-nucleation and lower oxidative DNA damage. Although this specialized subset of CM may explain the ~1% endogenous proliferation capacity in the adult myocardium, it remains unexplored whether heterogeneity of the stress-response gene expression changes among the larger majority of cell cycle-arrested CMs would uncover a sub-population that could be motivated to re-enter cell cycle.

We therefore undertook single CM nuclear RNA-seq of healthy and failing hearts, and uncovered the heterogeneity of CM transcriptomic stress-response. Using co-expression analysis, gene networks were constructed that pointed to key nodal lincRNA, which we further validated in vitro to regulate major de-differentiation and cell cycle genes. Our results altogether suggest that sub-populations of adult CMs exist, and possess a unique endogenous potential for cardiac repair by the targeting of key regulator lincRNA.

## Results

### Single nuclear RNA-seq of left ventricular CMs in vivo

Adult CMs are predominantly binucleated and undergo polyploidisation and multi-nucleation during heart failure^17^. To avoid confounding differences in comparing single cells with different number of nuclei, we reasoned that each single CM nucleus represents the simplest unit of transcription. We therefore performed single nuclear RNA-sequencing of PCM1^+^ (pericentriolar material 1) CM nuclei isolated from the left ventricles of transverse aortic constriction (TAC) mouse model of heart failure and Sham-operated control mice, as well as human end-stage failing hearts (non-ischemic dilated cardiomyopathy: DCM) and age- and sex-matched healthy controls. We focused on the left ventricle as it is the major site of pathological initiation of heart failure. PCM1 is an established marker of CM nuclei^3, 18, 19^ (Supplementary Fig. 1A, B). Since single-cell transcript detection stabilizes at low read depths^20–24^, we performed RNA-seq to an average depth of 8.5 ± 3.29 M mapped reads per sample, for a total of 359 single PCM1^+^ CM nuclei from both mouse and human left ventricles (Supplementary Table 1) using a well-published microfluidic single-cell transcriptomic platform^20–24^. Correlations showed good agreement of single nuclear expression with matched experimental pooled CM nuclei (*r* = 0.83 Sham, *r* = 0.86 TAC, Supplementary Fig. 1D, G), matched in silico pooled CM nuclei (*r* = 0.94 Sham, *r* = 0.98 TAC, Supplementary Fig. 1C, F), and even with matched bulk left ventricles (*r* = 0.61 Sham, *r* = 0.68 TAC, Supplementary Fig. 1E, H), which contain CM as well as other cell types such as fibroblasts and endothelial cells. In all cases, correlation values plateaued once we had sampled ~30 nuclei (Supplementary Fig. 1K–L), similar to saturation observed in previous single-cell RNA-seq reports^20–24^, demonstrating that our chosen sample size had sufficiently exceeded this saturation limit. A previous mouse RNA-seq paper using a similar TAC induced pressure overload mouse model at 8-week post TAC time point reported using a cutoff of at least fragments per kilobase per million mapped reads (FPKM) ≥ 3 (~1 copy per cell) in at least 1 heart to detect cardiac-relevant genes in bulk mouse heart tissue^25^. In view of potential noise issues in single nuclear RNA-seq, we set a more stringent criterion for genes to be expressed if it had at least FPKM ≥ 4 in at least 5 samples. In total, we achieved ~4.29 billion mapped reads (Supplementary Table 1) and identified a total of 4787 and 7642 genes expressed in Sham and TAC mouse CM nuclei respectively (Supplementary Data 1 and 2). Notably, previous whole tissue RNA-seq comparison of TAC vs. Sham mouse hearts reported a dramatic increase in the number of differentially expressed genes (1435 genes) in hearts at the 8-week post-TAC time point compared to 1-week post-TAC, consistent with much more extensive cardiac remodeling at 8-week and similar to the large increase in expressed genes we found at this same time point^25^.

To address any potential issue of technical variability in single nuclear RNA-seq, we performed several controls. First, we undertook technical replicates of the same nuclear RNA-seq samples using three independent library preparations and found good correlation (*r* = 0.99) across all three technical replicates (Supplementary Fig. 2A–C), reflecting a consistent library preparation procedure, and the absence of a batch effect in this regard. Second, we took the same nuclear RNA-seq samples with identical library preparation we had previously sequenced and performed sequencing again and found similarly good correlation (*r* = 0.94) (Supplementary Fig. 2D). Next, we loaded ERCC spike-in mix at pre-defined concentrations onto two separate microfluidic C1 chips and again recovered good correlation (*r* = 0.99) between single samples within the same chip (Supplementary Fig. 2E, F), and also across two independent C1 chips (*r* = 0.99, Supplementary Fig. 2G). Observed FPKM levels for the spike-in mix were consistent at expected concentrations (Supplementary Fig. 2H). Taken together, these controls excluded any significant technical variability in our single nuclear RNA-seq procedure.

### Core CM gene regulatory network

**Fig. 1:**
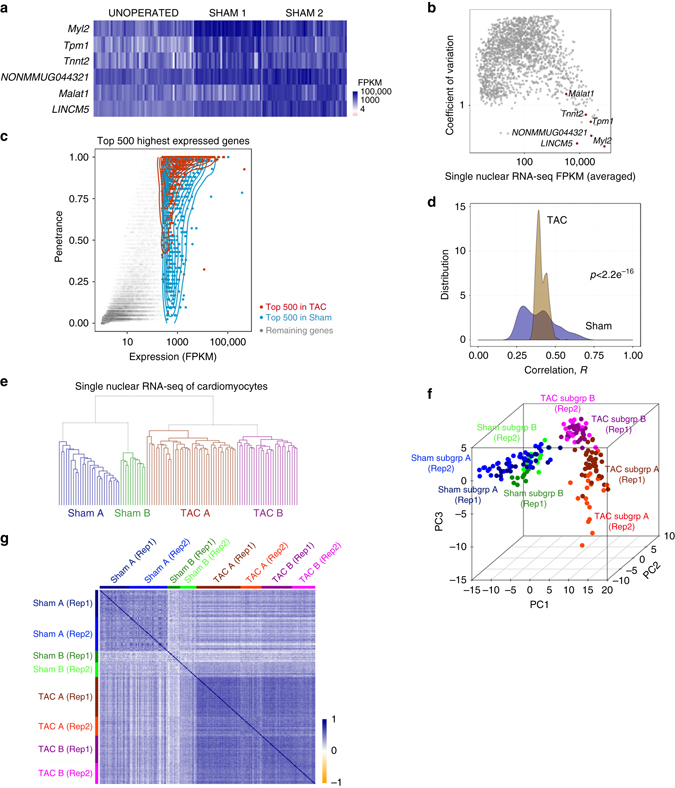
Single nuclear RNA-seq reveals CM heterogeneity. **a**, **b** Core cardiac genes that are most highly expressed in every CM nucleus (**a**) exhibit high expression with low coefficient of variation (**b**). **c** Highly expressed genes in TAC nuclei have higher penetrance than highly expressed genes in Sham nuclei. Spearman’s rank correlation (*r* = 0.90, *p* < 2.2e^−16^) shows good correlation between average expression level and penetrance. **d** Density distribution of correlation shows higher correlation in TAC nuclei than in Sham nuclei. *p* < 2.2e^−16^ from Mann–Whitney *U* test. **e**, **f** Unsupervised hierarchical clustering **e** and PCA **f** of single nuclear RNA-seq of CM reveal that CM nuclei accurately segregate into clusters specific to Sham or TAC subgroups (subgroup **a**, **b**) and is replicated across biological repeats (Rep) **f**. **g** Ranked Spearman correlation plot shows higher correlation in TAC nuclei than in Sham nuclei, which is replicated across biological repeats (Rep)

First, our single nuclear RNA-seq data set allowed us to define molecular markers that are present in every healthy CM nucleus. We identified 6 “core genes” that were the most highly expressed in every Sham nucleus, and also in healthy unoperated nuclei, with low coefficient of variation (CoV; Fig. 1a, b). We recognized that the consistent high expression specifically of *Tnnt2*, *Tpm1*, and *Myl2*, and not other previously assumed markers such as myosin heavy chain genes, imply their ideal suitability as markers for CM identity. Interestingly, the other three core genes were non-coding RNAs, reflecting a previously unappreciated abundance or function of these non-coding RNAs in CM nuclei.

### Heterogeneity and sub-populations of CMs in healthy and failing hearts

We next explored heterogeneity across samples. Instead of assessing the spectrum of expression level for each gene, we considered each sample categorically as either expressing or not expressing each gene; leading to a “penetrance” value for each gene, defined as the percentage of samples expressing the gene. In general, highly expressed genes were expressed in the vast majority of samples (Spearman ranked correlation *r* = 0.90, *p* < 2.2e^−16^) but we noted that this observation was more so in TAC than in Sham (Fig. 1c). Consistent with the notion that CMs responded to TAC stress by activating a unifying transcriptional program across individual nuclei, we found that among TAC nuclei there was a narrower distribution of correlation values than Sham (*p* < 2.2e^−16^ Mann–Whitney *U*-test, Fig. 1d). Furthermore, by using either unsupervised hierarchical clustering, principal component analysis (PCA), or ranked Spearman’s correlation, we consistently detected two distinct large subgroups of nuclei in Sham and TAC respectively, replicated in a further set of biological repeats (Fig. 1e–g).

**Fig. 2:**
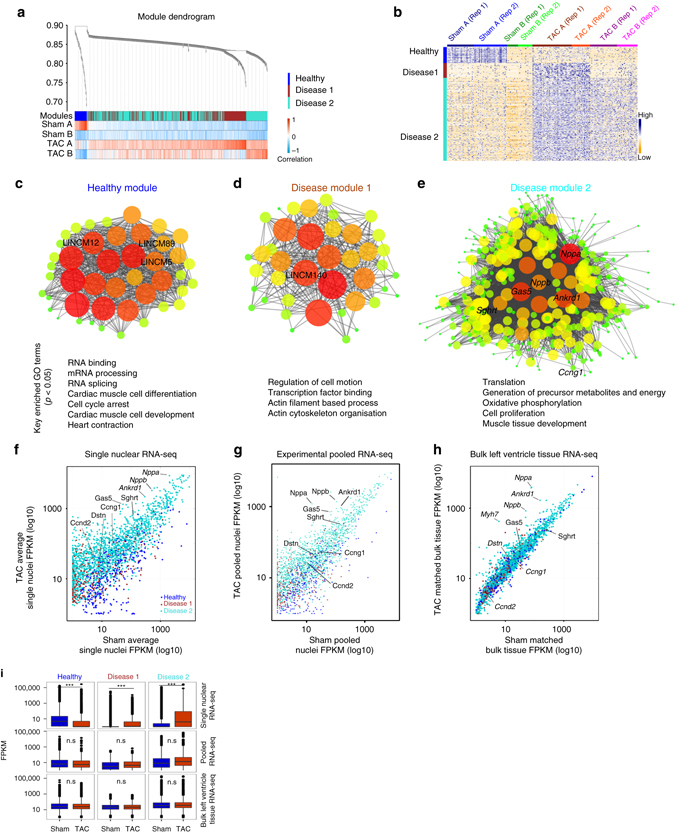
LincRNAs in nodal hubs of gene regulatory networks. **a**, **b** WGCNA identifies three distinct gene modules (Healthy, Disease 1 and Disease 2) (**a**) in Sham and TAC nuclei that represent expression signatures of specific Sham or TAC nuclear subgroups (**b**). **c**–**e** WGCNA reveals candidate lincRNAs in nodal hubs bearing the highest connectivity with other genes within the gene regulatory network modules. *Gas5* and *Sghrt* are in nodal hubs within disease module 2 (**e**) and highly correlated with expression of other genes in the network such as *Nppa*, *Dstn*, *Ccng1*, and *Ccnd2*. Size and color of bubbles represent strength and significance of connectivity. Key enriched gene ontology (GO) terms are listed for each module (*p* < 0.05 Fisher’s exact test). **f**–**h** Scatterplots showing the expression of genes from the 3 gene modules at the single-nuclear level (**f**), at pooled nuclei level (**g**) and matched bulk left ventricle tissue RNA-seq (**h**). **i** Significant differential expression of genes from the three gene modules between Sham and TAC samples is detected only by single nuclear RNA-seq, and not by pooled nuclei or bulk tissue RNA-seq

We performed weighted gene correlation network analysis (WGCNA)^26^ for the nuclear subgroups and identified highly correlated gene modules (Fig. 2a, b, Supplementary Data 3). Gene ontology analysis for the healthy module showed significant enrichment of genes for RNA binding, mRNA processing, RNA splicing, cardiac muscle cell differentiation, cell cycle arrest, cardiac muscle cell development and heart contraction (Supplementary Data 4, Fig. 2c). Disease module 1 contained apoptosis and autophagy genes, reflecting well-known pathways in heart failure^27^, and enrichment of genes in regulation of cell motion, transcription factor binding, actin filament based process, and actin cytoskeleton organization (Supplementary Data 4, Fig. 2d). Disease module 2 was enriched for genes in translation, generation of precursor metabolites, oxidative phosphorylation, response to oxidative stress, cell proliferation, and cardiac muscle tissue development, including well-known fetal reprogramming markers *Nppa* and *Nppb* (Supplementary Data 4, Fig. 2e). All three modules also contained important cardiac-expressed genes known to cause human dilated cardiomyopathy, hypertrophic cardiomyopathy, and congenital heart disease^28–30^, reflecting the overall physiological relevance of our gene modules to cardiac function (Supplementary Data 4).

Notably, genes in these modules now form a set of novel classifier markers because they are significantly differentially expressed in sub-populations of CM nuclei across Sham and TAC (Fig. 2f, i), otherwise masked by pooled and bulk tissue RNAseq approaches (Fig. 2g–i, Supplementary Data 5 and 6). Prominent exceptions to this remain classical fetal reprogramming genes such as *Myh7*, *Nppa*, and *Nppb* (Fig. 2h, Supplementary Data 6), which were stress-genes readily detectable even at bulk tissue level.

### Single nuclear RNA-seq of CM from human left ventricles

**Fig. 3:**
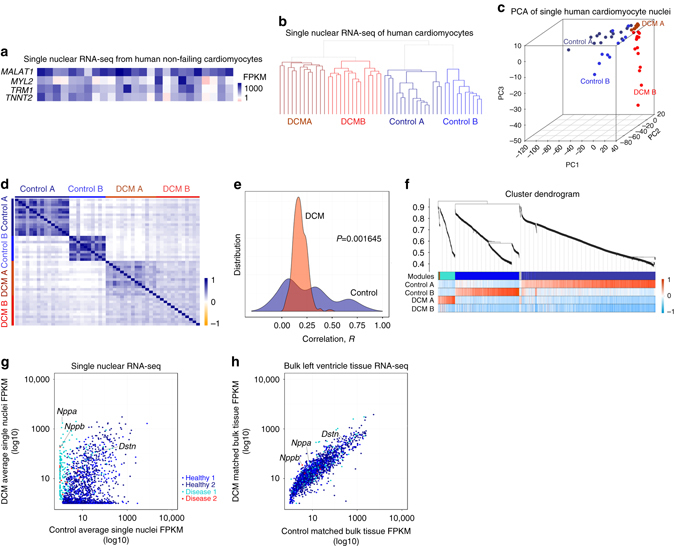
Human single cardiomyocyte nuclear RNA-seq. **a** Core cardiac genes in human CMs are similar to mouse. **b**–**d** Unsupervised hierarchical clustering (**b**), PCA (**c**) and Spearman correlation analysis (**d**) produced 2 distinct subgroups in each of control and dilated cardiomyopathy (*DCM*) nuclei. **e** Density distribution of correlation shows narrower distribution for DCM nuclei compared to control. *P* value from Mann–Whitney *U* test. **f** WGCNA identifies gene modules (healthy 1, healthy 2, disease 1, and disease 2) that are specific for DCM or control nuclear subgroups. **g**, **h** Classifiers from human gene modules show differential expression at single nuclear level (**g**), but not in matched bulk left ventricle RNA-seq (**h**)

We extended the same analysis to human CM nuclei from left ventricles of male DCM patients with end-stage heart failure compared with age-matched, male healthy controls. Remarkably, we found similar highly expressed core cardiac genes, nuclear subgroups, and reduced heterogeneity in DCM compared to controls (Fig. 3a–f). Gene Ontology analysis for gene modules (Supplementary Data 7 and 8) gave similar functional annotations as mouse (Supplementary Data 4). Differential expression of the dedifferentiation marker *DSTN* was detected at the single nuclear level, but not in bulk tissue RNA-seq (Fig. 3g, h), consistent with reports of increased DSTN protein in human DCM patient biopsies^31^.

### Heterogeneous cell cycle gene activation in stress-response

**Fig. 4:**
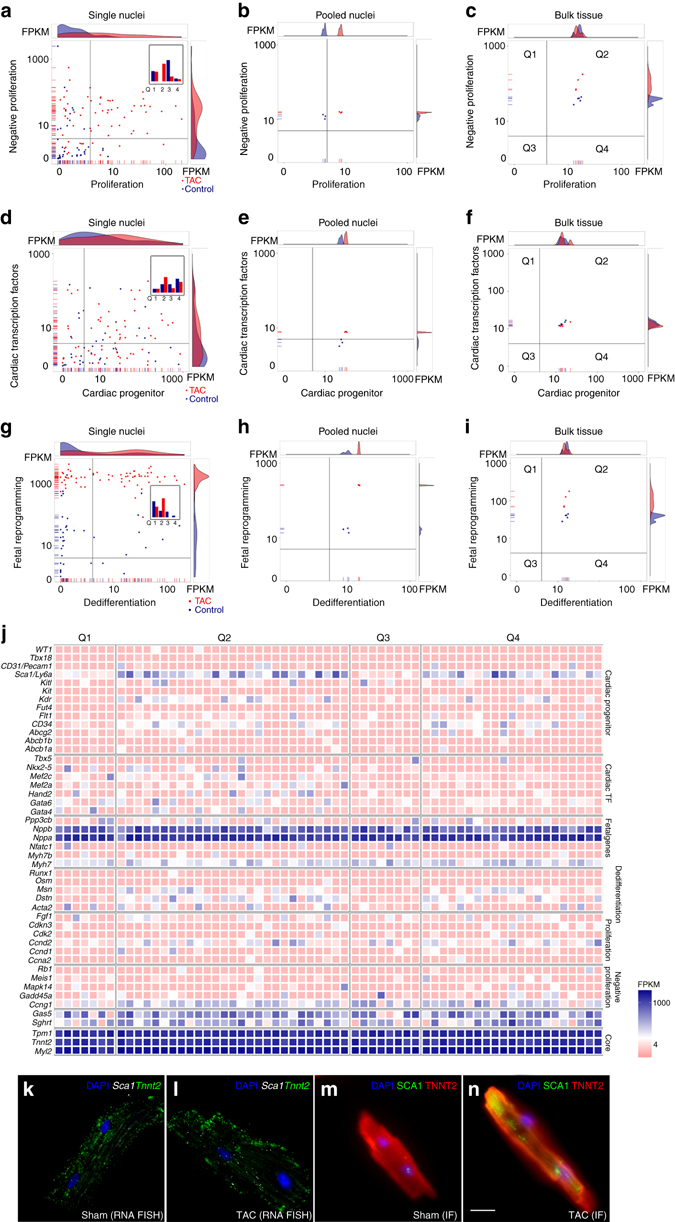
Quadrant analyses reveal sub-populations of CM. **a**–**c** Quadrant analysis for Proliferation vs. Negative regulators of proliferation genes identifies increased co-expression in individual TAC nuclei (Q2; 44.4%; *p* = 3.237e^−07^ Fisher’s exact test), only detectable by single nuclear RNA-seq (**a**), and not in pooled nuclei (**b**) or matched bulk left ventricle RNA-seq (**c**). *Inset*: histogram of nuclei distributed across quadrants. *Blue* represents Sham and *red* represents TAC nuclei. **d**–**f** Quadrant analysis for cardiac progenitor vs. cardiac transcription factor gene expression shows increased co-expression upon TAC stress in single CM nuclei **d** (Q2; 42.9%; *p* = 2.548e^−05^ Fisher’s exact test), again not detectable in pooled nuclei or bulk tissue RNA-seq (**e**, **f**). **g**–**i** Increased co-expression of fetal reprogramming genes and dedifferentiation markers under TAC stress only detected in single nuclear RNA-seq (**g**) (Q2; 58.73%; *p* = 0.001371 Fisher’s exact test) and not in non-single approaches (**h**, **i**). **j** High co-expression of cardiac progenitors, cardiac transcription factors, dedifferentiation, proliferation, and negative proliferation markers is confined to single nuclear TAC samples in Q2 and Q4. **k**, **l** Single molecule RNA FISH shows *Sca1* upregulation and co-expression of *Tnnt2* in isolated adult mouse CMs from TAC hearts **l** compared to Sham **k**. Number of *Sca1* ^+^ Sham CMs: 5/13; *Sca1* ^+^ TAC CMs: 38/55; all together from 2 Sham and 3 TAC biological replicates. **m**, **n** Immunofluorescence confirms increase in cell-surface SCA1 protein expression in TAC CMs (**n**) compared to Sham (**m**). Number of SCA1^+^ Sham CMs: 8/23; SCA1^+^ TAC CMs: 43/66; all together from 2 Sham and 3 TAC biological replicates. *Scale bar* represents 20 μm

Leveraging on the single nuclear resolution to give detailed insight into gene co-expression, we undertook “Quadrant Analysis” (Methods section) to compare expression profiles of sets of candidate genes, curated based on previously implicated importance for relevant CM biology. We started with “Proliferation” and “Negative regulators of Proliferation” markers in Sham and TAC mouse samples (Supplementary Table 4), and found a significant shift of nuclei from Sham in Q3 (Quadrant 3: not expressing either set of markers) to TAC in Q2 (Quadrant 2: co-expressing both sets of markers: 44.4%; *p* < 3.237e^−07^ Fisher’s exact test; Fig. 4a). This suggested that TAC nuclei activated proliferation gene transcription, and the same nuclei concurrently expressed negative regulators of proliferation acting as “molecular brakes” thus preventing successful cytokinesis. Among the candidate markers, *Ccnd2* and *Ccng1* were the major ones differentially expressed in the subgroup of TAC nuclei (Supplementary Fig. 3A, B). Of note, transgenic overexpression of *Ccnd2* promotes high rates of cardiomyocyte DNA synthesis and increased proliferation in adult mouse CMs^32, 33^, while overexpression of *Ccng1* promotes DNA synthesis, but inhibits cytokinesis leading to polyploidy and multinucleation^17^. Endogenous rate of division of pre-existing adult mouse CM is very low, with only a small increase during myocardial stress^1^. Accordingly, Q4 nuclei with high proliferation marker expression alone (6.4%, Q4; Fig. 4a) could be nuclei that retained the uninhibited potential for cytokinesis. Alternatively, there may be negative regulators in Q4 nuclei yet to be identified. Notably, only with the single nuclear resolution could we attain these results because the same population shifts were neither seen in pooled CM nuclei nor bulk left ventricle tissue (Fig. 4b, c).

### Early progenitor markers and markers of dedifferentiation

Next, we performed quadrant analysis for co-expression of cardiac progenitors and cardiac transcription factors, and observed upregulation of both markers in a large subset of TAC nuclei (Q2: 42.9%, Fisher’s exact test, *p* = 2.548e^−05^, Fig. 4d, Supplementary Table 4). This was again only detectable by single nuclear analysis, and not by pooled or bulk tissue analyses (Fig. 4e, f). *Sca1/Ly6a*, *Kdr*, and *CD34* as well as *Hand2*, *Nkx2-5*, *Mef2a*, and *Mef2c* were the major expressed markers in the subset of TAC CM (Fig. 4j). Endogenous *c-Kit* derived CMs were previously detected only at the very low percentage of ~0.03% in mouse hearts in vivo^34^. Among our samples, c*-Kit* was detected in only three mouse nuclei (0.83% of all nuclei). The cardiac progenitor marker *Isl1* was undetected in any sample. In contrast, high expression of *Sca1/Ly6a, Kdr, CD34* in failing adult CMs is surprising because these are markers of hematopoietic and endothelial progenitors that only give rise to very few adult CM in vivo^35, 36^. Moreover, *Sca1* ^+^ cardiac progenitor cells do not express cardiac contractile genes^35, 36^. We therefore assessed whether *Sca1* ^+^ nuclei were from progenitor cells or pre-existing adult CMs. In support of the latter, *Sca1* ^+^ nuclei co-expressed high abundance of core cardiac genes (*Tnnt2*, *Myl2*, *Tpm1*) (Fig. 4j). Furthermore, *Sca1* ^+^ nuclei made up a large proportion of TAC nuclei (Q2 and Q4: 81.0%; Fig. 4j) across biological replicates (70.3%; Supplementary Fig. 3C), contradicting the alternative possibility that these are progenitor-derived CMs. We confirmed low basal expression of *Sca1* RNA and cell-surface SCA1 protein expression in Sham CM and strong upregulation in TAC CMs by single molecule RNA FISH (fluorescent in situ hybridization) and immunofluorescence (Fig. 4k–n). Notably, we show that *Sca1* ^+^ CMs co-expressed *Tnnt2* RNA and protein (Fig. 4k–n), confirming their identity as adult CMs, and not fibroblasts or endothelial cells.

We further hypothesized that stressed nuclei exhibiting the fetal gene response would co-express dedifferentiation markers. Indeed, while TAC nuclei clearly had high expression of fetal genes, high co-expression with dedifferentiation markers was again only revealed by single nuclear analysis (Fig. 4g–i). Overall, key to the heterogeneous spectrum of stress-response was that upregulated co-expression of progenitor markers (*Sca1* and *Kdr*), dedifferentiation markers (*Dstn*, *Msn*, and *Actn2*), and cell-cycle genes (*Ccnd2* and *Ccng1*) were limited to the subset of TAC nuclei in Q2 and Q4 (Fig. 4j). This finding is important because it suggests that transcription of dedifferentiation and cell-cycle entry genes during stress-response in vivo could be controlled by common regulating factor(s) within each of these nuclei.

### Long intergenic non-coding RNA in nuclei of CMs

**Fig. 5:**
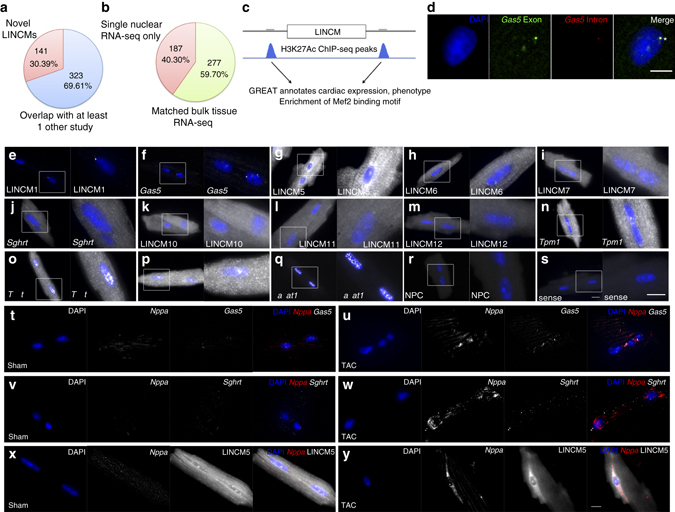
LINCM expression validated by single molecule RNA FISH. **a** Single nuclear RNA-seq identifies 141 novel lincRNAs in nuclei of CMs (*LINCMs*) that are not in current public databases (ENSEMBL and NONCODE) nor published cardiac transcriptome data sets. **b** Single nuclear RNA-seq identifies LINCMs that are not detectable in matched left ventricle bulk tissue RNA-seq, explained by the dilution of reads in cytoplasmic mRNA pool. **c** Active H3K27Ac enhancer chromatin regions proximal to LINCMs are enriched in MEF2 transcription factor binding motif and functionally annotated by GREAT analysis to have cardiac expression and phenotypes. **d** Sites of active transcription demonstrated by co-localization of exonic and intronic probes (*asterisk*) in nucleus. *Scale bar* represents 5 μm. **e**–**m** Single molecule RNA FISH validates the expression of LINCMs in isolated adult mouse CMs. **n**–**q** Positive controls for highly abundant core genes *Tpm1*, *Tnnt2*, *Myl2*, and *Malat1*. **r**, **s** Negative controls with no-probe control (NPC) (**r**) and sense probe (**s**) to confirm signal specificity. *Scale bar* represents 10 μm. **t**, **u** *Gas5* is upregulated in TAC CM and co-localizes with perinuclear *Nppa* transcripts. **v**, **w** *Sghrt* is upregulated and localizes to the cytoplasm of TAC CM. **x**, **y** LINCM5 is downregulated in TAC CM. *Scale bar* represents 10 μm

In effort to identify novel gene regulators in our nuclear RNA-seq data sets, we discovered a large number of long intergenic non-coding RNA in nuclei of CMs (LINCMs). Some of these were highly co-expressed with genes within our healthy and disease modules (Supplementary Data 3; Fig. 2c–f), raising the possibility that some LINCMs could play a regulatory role for coordinating the stress-response within gene modules. To ensure reliable annotation of LINCMs, we used Coding Potential Assessment Tool (CPAT)^37^ to rule out transcripts with coding potential. This led to a curated list of 464 LINCMs (Supplementary Data 9), of which 30.4% (141/464) were novel and 69.6% (323/464) were previously reported in public databases (ENSEMBL and NONCODE) or independent published cardiac transcriptome data sets (Fig. 5a)^25, 38–42^. We reasoned that we have detected more lincRNA because our RNA-seq was performed on nuclei instead of whole cells. Indeed, 40.3% (187/464) of our LINCMs were specifically detected only in our nuclear RNA-seq and not in matched bulk left ventricle RNA-seq (Fig. 5b). To ensure a fair comparison between the single nuclear and bulk tissue RNA-seq, we used either similar sequencing depths or ~8-fold higher sequencing depths in the bulk tissue, and the conclusion was the same: that our novel LINCMs were detectable only via the nuclear approach, and not in bulk tissue. It is hence possible that bulk tissue RNA-seq reads are predominantly occupied by the large pool of cytoplasmic mRNA, diluting out lowly expressed lincRNAs that are specifically nuclear retained, which are therefore not readily detected in bulk RNA-seq. Indeed, as an example, we found that LINCM6 is barely detectable in bulk left ventricle by reverse transcription PCR (Supplementary Fig. 4A, B) but have high abundance in our single nuclear RNA-seq, and confirmed to be nuclear localized by RNA FISH (Fig. 5h).

We explored the possibility of interactions between transcription factors and our list of LINCMs by performing motif analysis of empirical H3K27Ac ChIP-seq peaks demarcating active enhancer chromatin regions^43^ proximal to LINCMs loci. There was significant enrichment of cardiac transcription factor co-occupancy motifs^44^ at these loci (Fig. 5c, Supplementary Table 2). Notably, MEF2, a central transcription factor for cardiac development and myocardial stress-response^43^ was enriched in 57.1% of loci. To provide functional annotation of LINCM loci, Genomic Regions Enrichment of Annotations Tool (GREAT) analysis^45^ showed significant specific enrichment of cardiac expression and functions (Supplementary Table 3). Global correlation of expression levels between LINCM with nearby genes, including cardiac protein-coding genes, strengthened with increasing linear chromosomal distance from LINCM loci (Supplementary Fig. 4C), implying that LINCMs may act through distal regulatory interactions or long-range chromosomal looping interactions. Taken together, this suggests our LINCMs are biologically relevant to CM and could serve important epigenetic regulatory functions.

To ensure that our LINCMs exist in CMs and are not simply sequencing artifacts, we successfully validated 11 out of 12 candidate LINCMs by reverse transcription PCR (Supplementary Fig. 4A, B) and single molecule RNA FISH^46, 47^ in isolated adult CM (Fig. 5d–s) that concurrently demonstrated their sub-cellular localization patterns. Intronic and exonic probes co-localized at bright foci within the nucleus (Fig. 5d, asterisk), representing sites of active transcription^46^. Positive controls included highly abundant core cardiac genes *Tpm1*, *Tnnt2*, *Myl2*, and *Malat1* (Fig. 5n–q) and negative controls included no-probe control and sense probe controls (Fig. 5r, s). We confirmed that LINCM3 (also called *Gas5*) and LINCM9 (previously annotated *1810058i24Rik*, which we now call “Singheart”, *Sghrt*) were upregulated in TAC CMs, while LINCM5 was downregulated in TAC CMs as compared to Sham CMs (Fig. 5t–y). *Gas5* is located in the nucleus of Sham CMs (Fig. 5t) but is upregulated under TAC stress and co-localized with *Nppa* transcripts in the perinuclear regions of TAC CMs (Fig. 5u). *Sghrt* has low basal expression in nuclei and cytoplasm of Sham CMs (Fig. 5v) but is upregulated under TAC stress (Fig. 5w). Indeed, among all the lincRNA candidates in our foregoing network analysis, *Gas5* and *Sghrt* specifically occupied highly inter-connected nodal hubs within Disease module 2 (Fig. 2e), and stood out with the highest Eigengene-based connectivity kME, pointing to their potential key role as regulators of other genes within the same gene regulatory network.

### LincRNA regulate dedifferentiation and cell-cycle genes

**Fig. 6:**
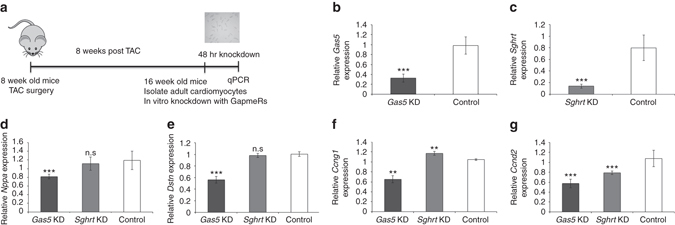
*Gas5* and *Sghrt* regulate co-regulated network gene expression. **a** Strategy to KD *Gas5* or *Sghrt* independently in isolated adult TAC CMs in vitro. *Cartoon* from openclipart.org, under a CC0 1.0 Universal license. **b**, **c** In vitro KD of *Gas5* or *Sghrt* in adult mouse CMs using GapmeRs is efficient and reproducible across biological replicates. *N* = 5 biological replicates. **d**–**g** *Gas5* KD in TAC CMs results in significant reduction of *Nppa*, *Dstn*, *Ccng1*, and *Ccnd2* expression. *Sghrt* KD in TAC CMs results in significant increase in *Ccng1* and reduction in *Ccnd2* expression. *N* = 5 biological replicates. Data represented as mean ± s.e.m. *p < 0.05, **p < 0.01, and ***p < 0.001

Our discovery of *Gas5* and *Sghrt* in key nodal hubs presented the testable hypothesis that they regulate co-expressed genes within the same gene regulatory network including cell cycle genes*: Ccng1* and *Ccnd2* and others: *Nppa* and *Dstn* (Fig. 2e; Supplementary Data 3). To functionally test this hypothesis, we performed knockdown (KD) of *Gas5* or *Sghrt* separately on isolated adult mouse CM (TAC-operated mice) using antisense LNA based GapmeRs and extracted RNA 48 h post KD (Fig. 6a). To ensure that reliable KD was achieved, we performed quantitative PCR (qPCR) and validated that *Gas5* and *Sghrt* were significantly reduced by 67.3% and 86.0% respectively (Fig. 6b, c; *Gas5* expression level after KD: 32.7% ± 8.29% s.e.m; *Sghrt* expression level after KD: 14.0% ± 3.50%; s.e.m.). For negative controls, we used both non-targeting negative control oligo as well as mock-transfected control.

KD of *Gas5* in adult TAC CMs significantly reduced the expression levels of *Nppa*, *Dstn*, *Ccng1*, and *Ccnd2* (Fig. 6d–g). Prior evidence show that *Gas5* accumulates upon growth arrest^48^, is expressed in many tissues including the heart^48^, and regulates apoptosis^49^ and proliferation^50^ in cancer cells. *Sghrt*, on the other hand, is a novel lincRNA with no previously described function. KD of *Sghrt* caused a significant increase in *Ccng1*, reduction in *Ccnd2*, but no significant change in *Nppa* or *Dstn* expression (Fig. 6d–g). Altogether, these confirm that nodal LINCMs (*Gas5* and *Sghrt*) regulate the transcriptional levels of genes in the same gene regulatory network.

## Discussion

Our single nuclear RNA-seq study of CMs from failing and non-failing mammalian hearts reveals for the first time, heterogeneity of the in vivo myocardial stress-gene response. We noted distinct sub-populations of CMs and uncovered gene regulatory networks specific for each sub-population, displaying specific sub-group upregulation of cell cycle, and de-differentiation genes in the disease stress response. We further identified LINCMs that occupy key nodal hubs in gene regulatory networks, and validated that KD of nodal LINCMs (namely, *Gas5* and *Sghrt*) leads to corresponding changes in the expression of co-regulated network genes, including those known to control CM cell cycle. Our findings suggest that nodal LINCMs may therefore act as key regulators of CM cell cycle during the endogenous myocardial stress response, and further work is warranted to investigate their direct effects on cardiac regeneration.

Other candidate regulators of CM proliferation have been previously reported. Conditional deletion of the homeodomain transcription factor *Meis1* in the postnatal mouse heart increased CM proliferation^11^. Postnatal inhibition of miR-15 family prolonged the proliferative capacity of neonatal CM^12^. Through a systematic screen with miRNA mimics, 2 inducers of CM proliferation, miR-199a and miR-590, were reported^13^. miR-99/100 and Let-7a/c have been reported to regulate the cardiac regenerative response in zebrafish and mouse hearts^15^. Hippo-deficient embryos had overgrown hearts with elevated CM proliferation^10^. Mitogens including neuregulin^51^, periostin^52^, and FGF1, in combination with p38αMAPK inhibition^53^, also promote adult CM cell cycle re-entry and completion of cytokinesis, although this effect may be restricted to a mono-nucleated subset of CM in rodents^16, 51, 52^. The low, but significant, degree to which each pathway is separately able to activate a small number of CM each time to undergo complete cytokinesis, has begged the question of whether this refers a single unique subset of CMs, or whether there are many subsets of CMs, each with unique pathways to activate cell cycle re-entry that are not co-linear. Our report of CM heterogeneity is consistent with a diverse spectrum of gene expression abundance from sample to sample. It may also be that the dominance of each pathway is stochastic and fluctuates in the lifetime of each CM, but certainly this notion is coherent with the teleological need for the heart to maintain cell-cycle arrest by employing as many pathways of inhibition as it needs. Still, our analysis has uncovered at least one subpopulation in which both cell cycle activators and inhibitors are co-activated during the disease stress-response.

The gene regulatory networks and LINCMs derived from our single nuclear RNA-seq now serve as an invaluable resource for identifying key endogenous regulators of cardiac regeneration. Meanwhile, the mitotic potential found in a substantial subset of adult CM in vivo raises the hope that targeting negative regulators of CM proliferation may one day lead to successful cardiac regenerative therapy.

## Methods

### Experimental animals

Ethical approval was from the National University of Singapore IACUC. Male C57BL/6 8-week post TAC and Sham-operated mice (16 weeks old) were used for all experiments.

### Single nuclear RNA-seq library preparation

Single nuclei were isolated from snap-frozen mouse and human left ventricle and processed by mechanical dissociation at 4000 Hz (4 × 20 s pulses) in Lysonator cartridges (SG Microlab devices) and counterstained with DAPI. CM nuclei were stained with PCM1 antibody (1:500, HPA023374, Sigma), secondary anti-rabbit Alexa 488 (1:500) or Alexa 568 antibody (1:500), and captured individually using C1 Single Cell Auto Prep system (10–17 uM mRNA seq chip, Fluidigm). Automated imaging of captured nuclei was performed on an inverted microscope (Olympus) with 10× objective (Olympus) and CCD camera (Axiocam MR3, Zeiss) to confirm the identity of wells containing only single PCM1^+^ CM captured. PCM1^+^ CM nuclear RNA-seq libraries were prepared using Nextera XT DNA sample preparation kit (Illumina). Each sample was sequenced with paired end 2 × 101 bp reads on HiSeq 2500 (Illumina).

### Human left ventricle samples

Human left ventricles were collected with a protocol approved by the Papworth (Cambridge) Hospital Tissue Bank Review Board and the Cambridgeshire Research Ethics Committee (UK). Written consent was obtained from all individuals according to the Papworth Tissue Bank protocol. DCM left ventricles were from patients undergoing cardiac transplantation for end-stage DCM^54, 55^. At the time of transplantation or donor harvest, whole hearts were removed after preservation and transported in cold cardioplegic solution (cardioplegia formula and Hartmann’s solution). Following analysis by a pathologist, left ventricle segments were cut and stored immediately in RNAlater (Ambion). Healthy normal left ventricles were from age-matched male individuals, through Papworth Hospital or Ethical Tissue (University of Bradford), governed by the UK Human Tissue Authority.

### Mouse surgery and isolation of mouse ventricular CM

TAC surgery was performed as previously described^56^. CM isolations were performed as previously published^57^ by enzymatic dissociation using perfusion buffer, 37 °C pre-warmed 40 ml enzyme solution (Collagenase II 0.5 mg/ml (Worthington), Collagenase IV 0.5 mg/ml (Worthington), and Protease XIV 0.05 mg/ml) at a rate of 2 ml/min. Enzymes were neutralized with fetal bovine serum (FBS) to final concentration of 5%. Cell suspensions were filtered through 100 μm nylon mesh cell strainers (Thermo Fisher Scientific) and allowed to settle by gravity. Calcium concentration was increased gradually to 1.0 mM. Cells were resuspended in plating medium containing M199 medium with glutamine (2 mM), BDM (10 mM), and FBS (5%), plated onto laminin-coated glass coverslips (#1, Thermo Fisher Scientific) and incubated for 1 h at 37 °C in a humidified environment with 5% ambient CO_2_. Non-attached cells were removed by gentle washing in PBS.

### Single molecule RNA FISH

Isolated CM adhered onto laminin coated #1 coverslips (ThermoScientific) were fixed for 10 min at r.t.p with Fixation Buffer (3.7% formaldehyde in PBS), washed twice in 1x PBS and permeabilized with 70% EtOH at 4 °C for at least an hour. RNA FISH was performed using 20-mer Stellaris Biosearch Probes for LINCMs and core genes conjugated to Quasar 670 or CAL Fluor Red 610. Briefly, cells were washed with Wash Buffer (10% formamide in 2x SSC) prior to overnight 37 °C hybridization with target probes (125 nM) in Hybridization buffer (100 mg/ml Dextran Sulfate, 10% Formamide in 2x SSC). After hybridization, cells were washed in Wash Buffer for 30 min at 37 °C, counterstained with DAPI (5 ng/ml in Wash Buffer) for 30 min at 37 °C, and washed in 2x SSC at r.t.p. Coverslips were transferred onto glass slides with mounting medium (Vectashield) and imaging was performed immediately on upright microscope (Nikon Ni-E) with 100x Objective (Nikon) on a cooled CCD / CMOS camera (Qi-1, Qi-2,Nikon).

For the notable exception of *Sca1*/*Tnnt2* RNA FISH co-staining, RNA FISH was performed using 50-mer ZZ ACD RNAScope probes due to the short unique sequence of *Sca1* available for probe design and high degree of homology to other members of *Ly6* family. Cells were fixed and permeabilized as above in 70% EtOH, washed in 1x PBS and 1x Hybwash buffer for 10 and 30 min respectively at r.t.p. prior to incubation with 1x Target Probe Mix at 40 °C for 3 h. Cells were washed thrice in 1x Hybwash at r.t.p, incubated in 1x Pre Amp Mix for 40 min at 40 °C, washed thrice in 1x Hybwash at r.t.p, incubated in 1 x Amp Mix for 30 min at 40 °C, washed twice in 1x Hybwash before incubation in 1x Label Probe Mix (Alexa Fluo 488, ATTO0550) at 40 °C for 25 min. Cells were washed thrice in 1x Hybwash in dark at r.t.p, counterstained with DAPI (5 ng/ml) prior to mount and imaging.

### Immunofluorescence

Isolated CM adhered onto coverslips were fixed in 4% formaldehyde and permeabilized with 0.5% Triton X for 10 min at r.t.p, prior to blocking in 5% BSA/PBS at r.t.p for 30 min. Cells were then incubated with primary antibodies diluted in 3% BSA/PBS overnight at 4 °C. Primary antibodies used include TNNT2 (1:100, ab8295, Abcam). SCA1 immunofluorescence was performed using two independent antibodies from two different companies SCA1 (1:50, E13 161–7, Abcam), SCA1 (1:100, AF1226, R&D) for technical validation and no Triton X was used for permeabilization to preserve cell-surface epitopes of Sca-1. Cells were washed thrice in 1x PBS, incubated in appropriate fluorescent secondary antibodies Donkey anti Rat Alexa Fluo 488, Donkey anti Goat Alexa Fluo 488 or Rabbit anti Mouse Alexa Fluo 568 and DAPI (5 ng/ml) for 60 min at r.t.p in dark. Cells were washed thrice in 1x PBS in dark before being mounted onto slides and imaged on an upright microscope Ni-E (Nikon).

### Knockdown of LINCMs

LNA GapmeRs were designed and ordered from Exiqon. Five different oligos were tested per LINCM for KD efficiency by qPCR at 48 h post transfection and the oligo with the best LINCM KD efficiency was used for subsequent experiments. Isolated TAC adult CMs were transfected with lipofectamine/GapmeR at a concentration of 100 nM and RNA extracted 48 h post transfection. Crucially, fetal reprogramming gene (*Nppa*) was highly upregulated (average ~27x) in TAC CM compared to Sham CM at the time of RNA harvest, indicating that during the short period in culture, the stress gene response remained intact in the isolated TAC cells. Negative control oligo with no known mRNA, lncRNA, miRNA targets in mouse or humans as well as mock-transfected cells (lipofectamine only) were used as negative controls. Five independent biological replicates were performed for each qPCR experiment. Each experiment had validated KD of target LINCM. Sequences of GapmeRs used are as follows: 5′-3′.

*Gas5* KD: AGAACTGGAAATAAGA

*Sghrt* KD: TTCGGAACTTGAAGGA

Negative control KD: AACACGTCTATACGC

### Real-time qPCR after knockdown of LINCMs

SuperScript III First-Strand Synthesis Reverse Transcriptase (Life Technologies) was used to reverse transcribe poly(A) RNA to cDNA. qPCR reactions were performed using SYBR Green master mix (SensiFAST, Bioline) in a LightCycler 480 machine (Roche). Threshold cycle (Ct) and melting curve measurements were determined by LightCycler 480 software. Each qPCR sample had at least three technical replicates on the same qPCR plate. *Rplp0* was used as housekeeping gene and Ct values were normalized to mock-transfected (no oligo, lipofectamine only) samples. *P* values from Student’s *t*-test and error bars represent s.e.m. Five biological replicates of adult isolated TAC CMs were used for qPCR analysis of each gene. Primers used are listed in Supplementary Table 5.

### Sequencing libraries QC

We used well established quality-control tools such as CASAVA version 1.8.2 (Illumina), FASTQC (Babraham Bioinformatics) and Trimmomatic^58^ to filter raw reads. Filtered reads were aligned to mouse genome (Mus Musculus) mm9 assembly using mapping software, Tophat v2.0.9 with Bowtie2 using default settings^59, 60^. We provided mm9 ensembl 65 (version 1) GTF annotation to Tophat for mapping with –G option. To ensure only high quality libraries are used for analysis, single nuclear RNAseq samples with <40% mapping were excluded from subsequent downstream analyses. Transcript expression levels were calculated in FPKM by turning on fragment bias correction parameter (-b) and multi-read correction (-u) using Cufflinks v2.1.1^61^. We applied a stringent expression threshold by regarding transcript with FPKM lower than 4 to be non-expressing. Only genes that were expressed with FPKM ≥ 4 in at least 5 samples were considered for our subsequent analyses.

### Core cardiac genes discovery

Genes in each sample were sorted based on FPKM values from highest FPKM to lowest FPKM. Each gene was assigned a rank based on the sorted order. The gene with the highest FPKM was assigned a rank of 1. We defined core cardiac genes as genes that were to found to be expressed in all Sham-operated nuclei at FPKM ≥ 4 and displayed ordered rank within top 500 in all samples.

### Coefficient of variation vs average FPKM plot

We calculated the CoV, also known as normalized s.d. We defined CoV as the ratio of s.d. of FPKM value and mean FPKM value across all samples for each condition (Sham or TAC). CoV vs Average FPKM scatterplot of all expressed genes was generated with each point representing a single gene.

### Hierarchical clustering and expression heatmap

We used custom R function hclust to hierarchically cluster the samples based on the pearson correlations between samples. The hierarchical dendrogram was cut at a height of 0.75. This resulted in four branches of samples, which we defined as 4 distinct subgroups of cardiomyocytes, i.e., Sham A, Sham B, TAC A, and TAC B. The hierarchical tree was visualized using R package A2R, where each of the four sub-group was colored differently for visualization purpose. Expression heatmaps represented row-scaled log_2_ (FPKM + 1) values, where high intensity blue represents high expression while high intensity yellow represents low expression. The resulting subgroups were cross-validated using PCA. We transformed the FPKM values of each gene to have 0 mean and unit variance across all samples in order to compare variability patterns across genes with different overall abundance in the population. We used custom R function prcomp to perform PCA analysis. The largest three principal components are visualized in a three-dimensional scatterplot using R-package scatterplot3d version 0.3.35^62^. To confirm the presence of 4 subgroups, we also calculated a correlation matrix based on the log_2_ (FPKM + 1) values, and visualized the correlation, *r* value in a correlation heatmap.

### Correlation density

We calculated pairwise correlation between each sample in each condition (Sham and TAC). In order to assess the distribution of the correlation value, we plotted density plot, where each condition is colored differently. To test for significant changes in distributions of correlation between Sham samples and TAC single nuclear RNA-seq samples, we used Mann–Whitney U 2-sided test as we do not assume normal distribution of correlation in single nuclear RNA-seq or any particular direction of change.

### Saturation analysis

Using samtools version 0.1.19, saturation analysis was performed by randomly sub-sampling different number of reads from individual sample, and re-calculating the FPKM value for each genes. The process of sub-sampling was repeated until there were at least 10 subsampled data sets per point with increasing library size.

### Correlation saturation analysis

We randomly selected a pre-defined number of samples out of all available single-nuclear RNAseq samples to calculate average FPKM expression levels per gene. The average expression values were used to calculate coefficient of correlation with bulk tissue and pooled nuclei RNAseq expression level. We used pre-defined sets of 2, 5, 10, 15, 20, 25, 30, and 35 samples from the single-nuclear RNAseq samples with 10 replicates per set.

### In silico pooled cardiomyocyte nuclear RNAseq

Using samtools version 0.1.19, we pooled all of the mapped reads from the Sham and TAC single-nuclear RNAseq samples into Sham and TAC pooled nuclei respectively. 8 M reads (amount equivalent to average mapped reads in Sham samples in Batch 1) were subsampled randomly from Sham pooled nuclei, and 6 M reads (amount equivalent to average mapped reads in TAC samples in batch 1) were subsampled randomly from TAC pooled nuclei to generate pooled nuclei library with matched sequencing depth. 60 aggregated in silico pooled RNAseq samples were generated each for Sham and TAC to calculate average FPKM per gene for comparisons with single nuclear RNAseq and matched bulk tissue RNAseq.

### Weighted gene correlation network analysis

Using WGCNA^26^, we started the construction of a signed weighted correlation network by computing pairwise correlations between all genes across all single-nuclear RNAseq samples. Next, we chose soft thresholding power (*β* = 6), in constructing an adjacency matrix using the formula, *a*_ij_=(0.5+0.5×*s*_ij_)^*β*^, where *a*_ij_ is defined as weighted correlation and *s*_ij_ is defined coefficient correlation between gene_i_ and gene_j_. We choose the power (*β* = 6), which is the lowest power for which the scale-free topology fit index curve flattens out upon reaching a high value of 0.98. Using the adjacency matrix computed in the previous step, topological overlap was calculated to measure the network interconnectedness in a robust and biological meaningful way. The topological overlap was utilized to group highly correlated genes together using average linkage hierarchical clustering. Modules were defined as the branches obtained by cutting the hierarchal tree using Dynamic Hybrid Tree Cut algorithm^63^. We defined the first principle component of a module as module eigengene, which is representative of the expression profile in each module. Genes in each module were removed if the correlation between the gene and module eigengene (kME) is <0.3. If a detected module did not have at least 5 genes with eigengene connectivity (kME) at least 0.5, the module was disbanded and its genes were unlabeled and returned to the pool of genes to await module detection. Modules whose eigengenes were highly correlated (correlation above 0.75) were merged. Construction of signed gene network and identification of modules were performed using R function, blockwiseModules with following parameters: soft thresholding power = 3, minimum module size = 15, mergeCutHeight = 0.25, corType = “Pearson”, networkType = “signed”, TOMType = “signed”, minCoreKME = 0.5, and minKMEtoStay = 0.3.

### External clinical traits and hub gene identification

To identify modules that were significantly correlating with subgroups, we computed correlation of eigengenes of each module with all subgroup and picked the most significant associations as subgroup-specific modules. For visualization purpose, the correlation values are presented in a table matrix and color-coded based on the correlation values. In addition, we also computed gene significance (GS), defined as the correlation of each gene with each subgroup^26^. We calculated module membership (MM), defined as the correlation between module eigengene and gene expression profile. GS and MM are important because they help in the identification of genes with high significance for each subgroup and high module membership in each subgroup-specific module. Module membership is highly correlated to the intramodular connectivity, k_ME_. Highly connected intramodular hub genes tend to have high module membership values to the respective module^26^.

### Exporting modules to cytoscape for network visualization

We used R function exportNetworkToCytoscape to export the gene network for healthy, disease 1 and disease 2 modules to Cytoscape.

### Discovery of LINCMs

We used Cufflinks version 2.1.1 to perform novel transcript discovery in each nuclei after masking out all protein-coding genes, with default parameters. All of the predicted assemblies from all nuclei were merged using Cuffmerge version 2.1.1. The predicted transcripts in the merged assembly were checked for coding potential with CPAT^37^. CPAT uses a logistic regression model built with four sequence features for protein-coding potential prediction, including open reading frame size, open reading frame coverage, Fickett TESTCODE statistic, and hexamer usage bias. CPAT reports the protein-coding probability score in the range between 0 and 1, but the optimum probability cutoff varies with different organism. For mouse, we used optimum cutoff determined from TG-ROC (coding probability > 0.44) to classify our candidate lincRNA as coding or non-coding RNA. After filtering transcripts with coding potential, we looked for overlap between our predicted assembly and publicly available lncRNA databases such as NONCODE mm9 version 4 and GENCODE mm9 version M1. Next, we filtered away transcripts shorter than 200 bp. Lastly, we exclude transcripts with FPKM < 4 in <5 samples. In order to ensure the reproducibility of results, we repeated the above steps in the second replicate of single-nuclei samples. Only those transcripts discovered in both batches of sequencing were retained in subsequent analysis.

### Quadrant analysis

We selected gene markers for a category of interest based on current literature of cardiomyocyte biology, and used scatterplots to perform pairwise comparisons of expression levels between two groups of genes, x and y. FPKM of 4 was used as a threshold to divide each axis, resulting in 4 quadrants, namely as Q1 (high expression of y genes, low expression of x genes), Q2 (high expression of both x and y genes), Q3 (low expression of x and y genes), and Q4 (low expression of y genes and high expression of x genes). After defining the sample distribution in each quadrant, we performed pairwise differential expression analysis between samples of different quadrants to look for enriched genes within each quadrant. For human quadrant analyses, we included 4 additional DCM patients and 2 additional controls to make a total of 5 DCM patients and 3 controls.

### Differential expression analysis for quadrant analysis

Differential expression analyses between quadrants were performed using exact test in R version 3.0.0 DESeq version 1.12.1^64^.

### Data availability

Sequence data that support the findings of this study have been deposited in NCBI SRA SRP049944, under the BioProject code PRJNA264588. The data that support the findings of this study are available from the authors on reasonable request.

## Electronic supplementary material

Supplementary Information

Dataset 1

Dataset 2

Dataset 3

Dataset 4

Dataset 2

Dataset 6

Dataset 7

Dataset 8

Dataset 9
