# Supplementary material for: Single cardiomyocyte nuclear transcriptomes reveal a lincRNA-regulated de-differentiation and cell cycle stress-response in vivo
Source: Nat Commun. 2017 Aug 9;8:225. doi: 10.1038/s41467-017-00319-8 (PMC5548780; doi:10.1038/s41467-017-00319-8)
Supplement: Supplementary file 1 — Supplementary Information [file 41467_2017_319_MOESM1_ESM.pdf]

File Name: Supplementary Information
Description: Supplementary Figures and Supplementary Tables.

File Name: Supplementary Data 1
Description: Genes expressed in Sham nuclei.

File Name: Supplementary Data 2
Description: Genes expressed in TAC nuclei.

File Name: Supplementary Data 3
Description: Genes expressed in modules of mouse nuclei.

File Name: Supplementary Data 4
Description: Gene Ontology annotations of modules in mouse nuclei.

File Name: Supplementary Data 5
Description: Differential expression between Sham and TAC (Pooled Nuclei).

File Name: Supplementary Data 6
Description: Differential expression between Sham and TAC (Bulk Tissue).

File Name: Supplementary Data 7
Description: Genes expressed in modules of human nuclei.

File Name: Supplementary Data 8
Description: Gene Ontology annotations of modules in human nuclei.

File Name: Supplementary Data 9
Description: List of LincRNAs in Nucleus of Cardiomyocytes (LINCMs).

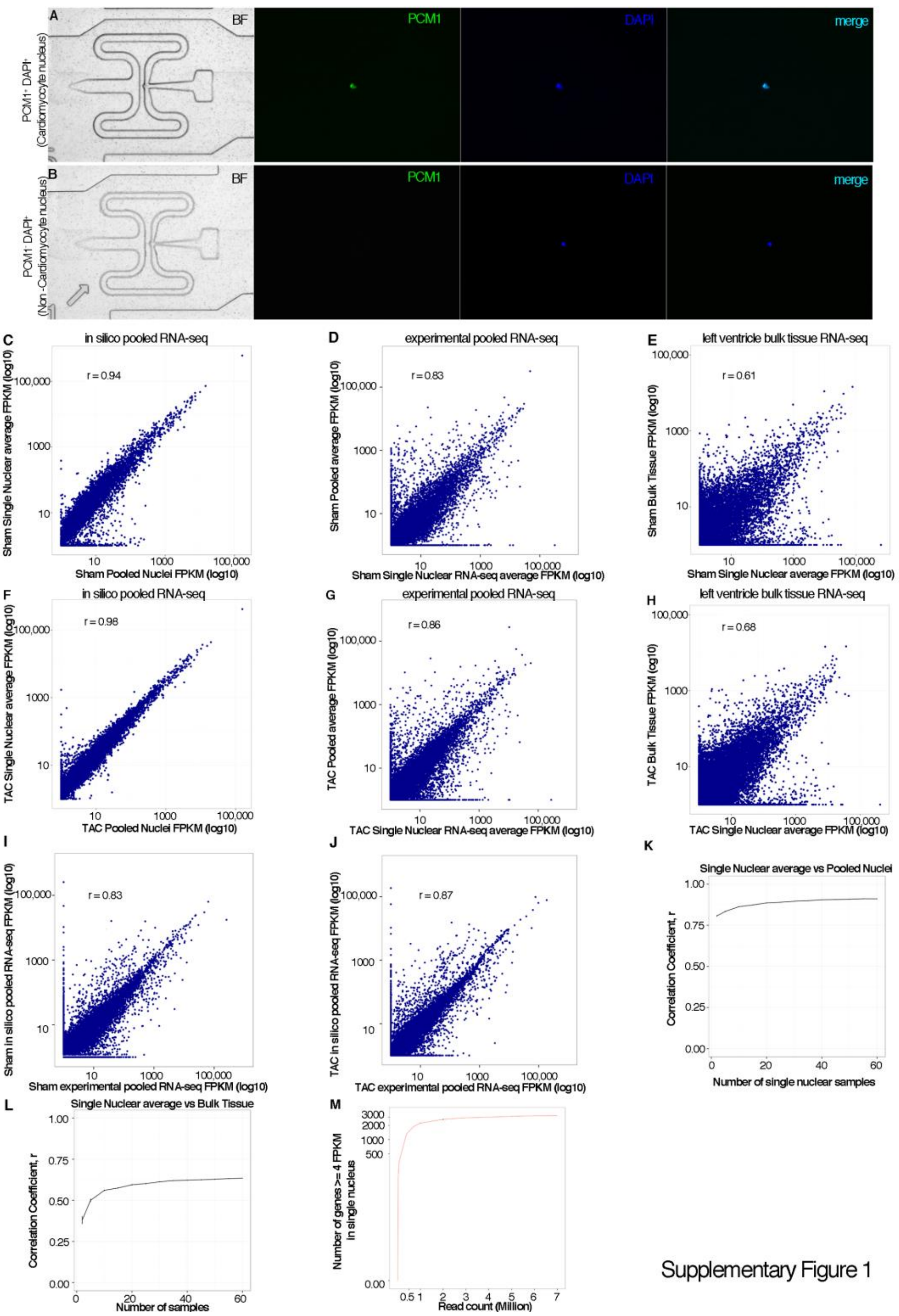

Supplementary Figure 1

Supplementary Fig. 1. Quality control assessment of single nuclear RNA-seq by correlation and saturation analyses

A-B, Representative image of a single CM nucleus (PCM1^{+}/DAPI^{+}) (**A**) and non-CM nucleus (PCM1^{-}/DAPI^{+}) (**B**) captured in microfluidic chamber of a Fluidigm C1 chip for single nuclear RNA-seq. **C-E,** Correlation plots

comparing averaged single nuclear RNA-seq to in silico pooled nuclei (**C**), to experimental pooled RNA-seq (**D**) and to matched bulk left ventricle tissue RNA-seq (**E**) in Sham. **F-H,** Correlation plots comparing averaged single nuclear RNA-seq to in silico pooled nuclei (**F**), to experimental pooled RNA-seq (**G**) and to matched bulk left ventricle tissue RNA-seq (**H**) in TAC.

I-J, Good correlations between experimental pooled RNA-seq and in silico pooled RNA-seq in either Sham (**I**) or in TAC (**J**). **K-L,** Correlations against increasing sample number shows that saturation of correlation was achieved at ~30 nuclei, consistent with previous single cell RNA-seq reports. **M,** Saturation analysis shows that our average sequencing depth of 8.5 million ± 3.29 s.d. reads per nucleus exceeds saturation at ~2 million reads, which demonstrates we have sufficient read depth per sample.

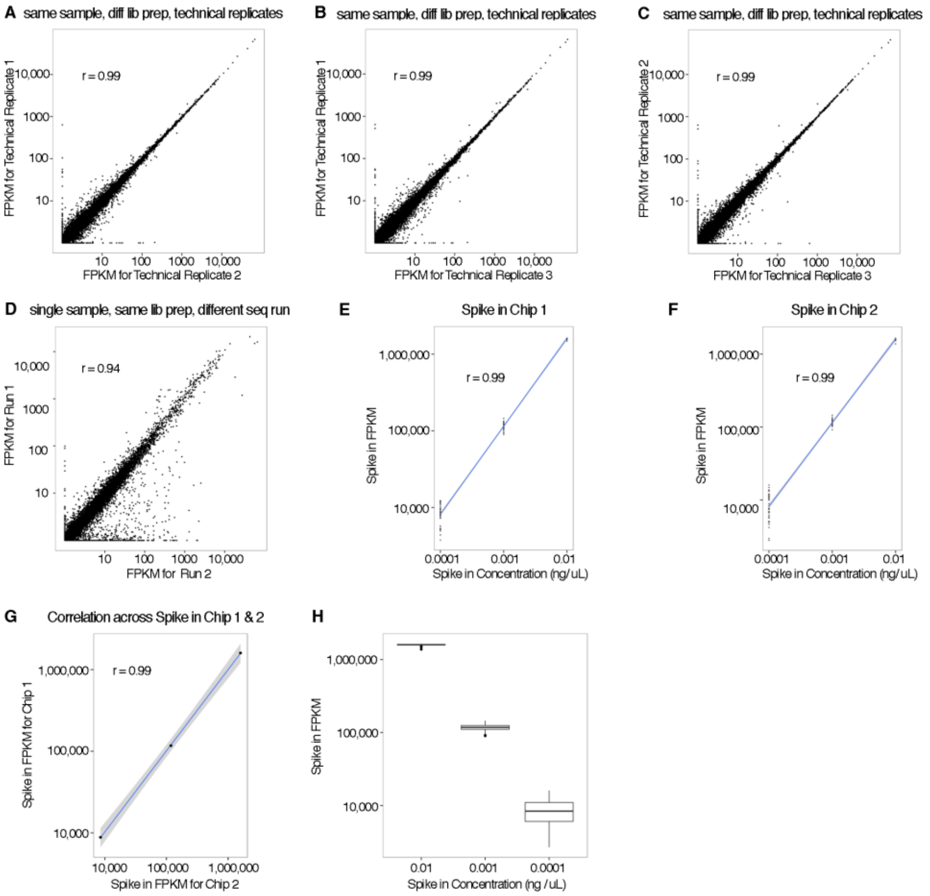

Supplementary Figure 2

Supplementary Fig. 2. Additional Quality Control assessment of technical variability of single nuclear RNA-seq using spike ins

A-C, High correlations observed for technical replicates of same nuclear RNA-seq sample prepared with different library preparations suggest minimal technical variability from library preparation process. **D,** High correlation observed for re-run of same nuclear RNA-seq sample with same library preparation after single freeze thaw cycle on independent HiSeq machine suggest minimal technical variability from sequencing platform. **E-F,** High correlations observed between samples within same C1 chip suggest minimal well to well variability in either chip 1 (**E**) or in chip 2 (**F**). **G,** High correlations observed between samples across two independent C1 chips suggest minimal well to well technical variability across independent experiments. **H,** Good agreement between expected FPKM values and actual spike in concentrations with minimal variability.

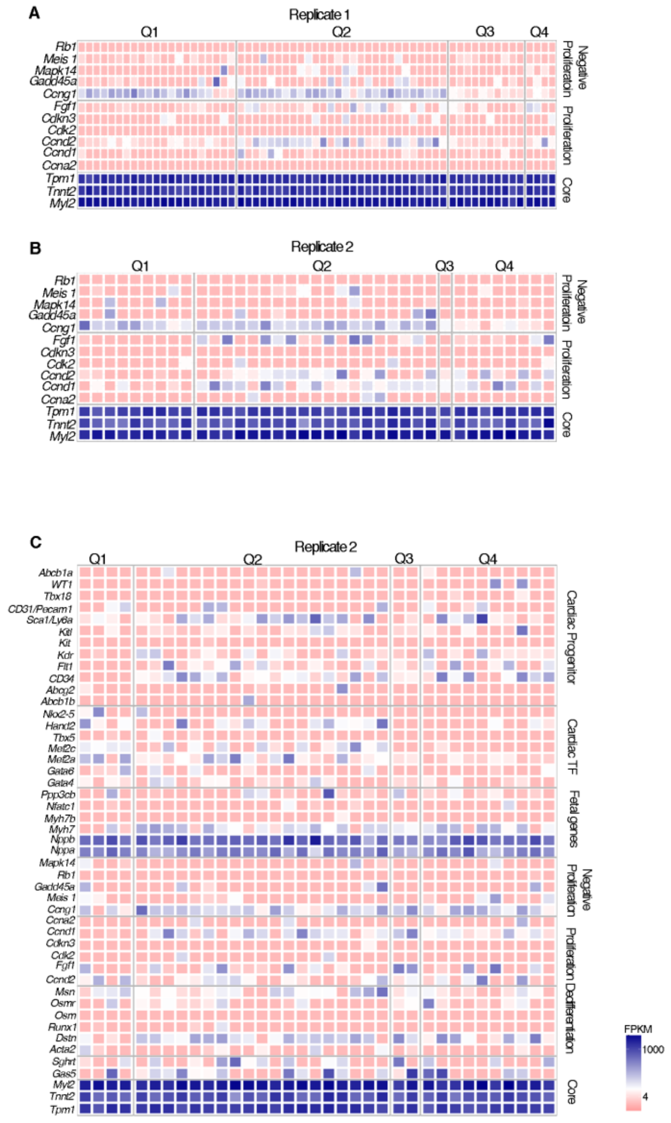

Supplementary Figure 3

Supplementary Fig. 3. Quadrant Analyses show that gene co-expression in TAC nuclei is highly reproducible across independent biological replicates.

A-B, Gene expression heatmap of TAC nuclei in Quadrants for Proliferation vs Negative Regulators of Proliferation genes identifies *Ccng1*, *Ccnd2*, *Ccnd1*

and *Fgf1* as major contributors in both biological Replicates 1 and 2. **C,** Gene expression heatmap of TAC nuclei in Quadrants for Cardiac progenitor vs Cardiac transcription factor genes in Replicate 2 shows re-expression of *Sca1*, *Kdr* and *CD34* progenitor markers in TAC (as in Replicate 1, **Figure 3J**).

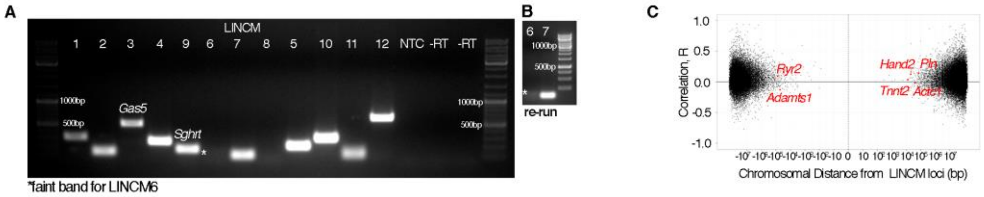

Supplementary Figure 4

Supplementary Fig. 4. Validation of LINCMs in heart by RT-PCR

A-B, Successful amplification of LINCMs by RT-PCR on DNAse treated RNA to exclude genomic DNA contamination (**A**). Re-run of LINCM6* shows a faint band of correct size on a separate gel (**B**). Products were gel extracted and

Sanger sequenced to confirm identity. **C,** Correlation of gene expression increased with increasing linear chromosomal distance away from LINCM loci. Cardiac relevant genes highlighted in red.

..

Supplementary Table 1. Statistics of RNA sequencing data generated in this study

Sample	Number of cDNA libraries sequenced and passed QC	Average number of mapped reads per sample	Total number of mapped reads per group
Mouse Replicate 1 Sham single nuclear	37/50 (74%)	6.6M	244M
Mouse Replicate 1 TAC single nuclear	64/64 (100%)	5.5M	352M
Mouse Replicate 2 Sham single nuclear	49/50 (98%)	9.3M	456M
Mouse Replicate 2 TAC single nuclear	39/40 (97.5%)	10.2M	401M
Mouse Unoperated single nuclear	54/54 (100%)	7.0M	379M
Mouse Bulk LV matched to replicate 1 Sham	1/1 (100%)	51M	51M
Mouse Bulk LV matched to replicate 1 TAC	1/1/ (100%)	55M	55M
Mouse Bulk LV matched to replicate 2 Sham	1/1 (100%)	16.9M	16.9M
Mouse Bulk LV matched to replicate 2 TAC	1/1 (100%)	12.9M	12.9M
Mouse Bulk LV unoperated	1/1 (100%)	18.3M	18.3M
Mouse Sham operated Bulk LV replicate	5/5 (100%)	16.8M	84M
Mouse TAC operated Bulk LV replicate	4/4 (100%)	17.2M	68M
Human Control Individual 1 single nuclear	25/31 (80.65%)	17.5M	437.8M
Human Control Individual 2 single nuclear	16/24 (66.67%)	9.7M	155M
Human Control Individual 3 single nuclear	7/7 (100%)	6.0M	42.2M
Human DCM Individual 1 single nuclear	26/27 (96.29%)	9.0M	234.2M
Human DCM Individual 2 single nuclear	13/19 (68.4%)	11.4M	148.5M
Human DCM Individual 3 single nuclear	13/26 (50.0%)	8.0M	104.1M
Human DCM Individual 4 single nuclear	6/15 (40%)	4.75M	28.5M

Human DCM Individual 5 single nuclear	10/22 (45%)	8.26M	82.6M
Human Bulk LV matched to control 1	1/1 (100%)	95.2M	95.2M
Human Bulk LV matched to control 2	1/1 (100%)	118.9M	118.9M
Human Bulk LV matched to control 3	1/1 (100%)	89.9M	89.9M
Human Bulk LV matched to DCM 1	1/1 (100%)	111.1M	111.1M
Human Bulk LV matched to DCM 2	1/1 (100%)	114.6M	114.6M
Human Bulk LV matched to DCM 3	1/1 (100%)	118.1M	118.1M
Human Bulk LV matched to DCM 4	1/1 (100%)	103.9M	103.9M
Human Bulk LV matched to DCM 5	1/1 (100%)	163.4M	163.4M

Supplementary Table 2. Top 50 Transcription Factor Co-occupancy Motifs identified in active chromatin H3K27Ac peaks within 100kb upstream and downstream of LINCMs

Rank	Name	Family	Score	Binding Range	P value
1	V$EVI1_04	GATA_DIMER	10.2591	1000	$5.91E^{-14}$
2	V$NKX62_Q2	NKX	8.57362	1160	$7.22E^{-09}$
3	V$FOXD3_01	FOX	8.34411	3040	$2.82E^{-08}$
4	V$SOX17_01	SOX	8.27772	2320	$4.14E^{-08}$
5	V$HELIOSA_02	HELIOS	8.05285	2600	$1.47E^{-07}$
6	V$HMGIY_Q3	HMGIY	8.00462	3600	$1.92E^{-07}$
7	V$CEBPGAMMA_Q6	CEBP	7.84755	4240	$4.46E^{-07}$
8	V$AMEF2_Q6	**MEF2**	7.628	4600	$1.39E^{-06}$
9	V$IRF_Q6_01	IRF	7.568	920	$1.89E^{-06}$
10	V$TEF_Q6	TEF	7.5025	960	$2.61E^{-06}$
11	V$CDX_Q5	CDX	7.42553	2200	$3.81E^{-06}$
12	V$BLIMP1_Q6	BLIMP1	7.31585	2680	$6.45E^{-06}$
13	V$OCT1_Q5_01	OCT	7.02966	3320	$2.41E^{-05}$
14	V$HNF4_Q6_02	ERE	6.97927	600	$3.01E^{-05}$
15	V$CIZ_01	AAAAA	6.62923	3000	0.000132
16	V$TBP_01	TATA	6.5954	1720	0.000151
17	V$HNF6_Q6	HNF6	6.23052	2200	0.00061
18	V$SZF11_01	SZF11	6.03982	3120	0.0012
19	V$LPOLYA_B	POLYA	5.99302	1040	0.00141
20	V$LEF1_Q2	LEF	5.96924	2120	0.00152
21	V$GATA2_03	GATA	5.96339	400	0.00155
22	V$ELF1_Q6	ETS	5.94841	1200	0.00163
23	V$HSF1_Q6	HSF	5.83068	960	0.00241
24	V$HNF1_Q6	HNF1	5.60948	1920	0.00481
25	V$PAX4_02	PAX	5.58008	3200	0.00525
26	V$POU1F1_Q6	POU	5.55822	600	0.00561
27	V$NFY_Q6_01	CAAT	5.52428	3520	0.0062
28	V$DMRT7_01	DMRT	5.48639	2000	0.00692
29	V$FAC1_01	FAC1	5.4664	5000	0.00733
30	V$STAT5A_04	STAT	5.43804	1640	0.00795
31	V$CART1_01	CART1	5.43268	1840	0.00807
32	V$RREB1_01	RREB	5.37239	1440	0.00957
33	V$MYOD_Q6	EBOX	5.34758	1320	0.0102
34	V$UF1H3BETA_Q6	SP1	5.33788	2960	0.0105
35	V$LYF1_01	LYF1	5.22372	1640	0.0143
36	V$TCF11_01	AP1	5.18948	4240	0.0157
37	V$BRN2_01	BRN2	5.1835	2160	0.0159
38	V$BARBIE_01	BARBIE	5.13604	2560	0.018
39	V$ATATA_B	ATATA	5.1188	2360	0.0188
40	V$PBX1_01	PBX	5.11423	640	0.019
41	V$AR_Q2	AR	5.10301	440	0.0196

42	V$HAND1E47_01	HAND1E47	5.07716	960	0.0209
43	V$LHX3_01	AT_RICH	5.07506	2400	0.021
44	V$MRF2_01	MRF2	5.0508	5000	0.0223
45	V$CDC5_01	CDC5	5.01918	1160	0.0241
46	V$AP2_Q6_01	AP2	4.92176	3400	0.0304
47	V$TST1_01	TST1	4.90303	4640	0.0318
48	V$AIRE_02	AIRE	4.90226	840	0.0318
49	V$LMAF_Q2	LMAF	4.89595	1480	0.0323
50	V$TITF1_Q3	TITF1	4.68556	1040	0.0516

Supplementary Table 3. GREAT analysis of active chromatin H3K27Ac peaks in vicinity of LINCMs identifies expression and phenotype in mouse heart and cardiac relevant miR binding site motifs.

MGI Phenotype	Binom Raw P value	Binom FDR Q value	Binom Fold enrichment
poor circulation	$1.18E^{-08}$	$4.3117E^{-05}$	6.307304
**decreased cardiac muscle contractility**	$6.39E^{-08}$	0.000116803	2.432103
**abnormal cardiac muscle contractility**	$2.41E^{-07}$	0.000293503	2.226063
**myocardial trabeculae hypoplasia**	$2.96E^{-07}$	0.000270137	3.449798
**abnormal trabecula carnea morphology**	$4.49E^{-07}$	0.000364571	2.823403
**myocardium hypoplasia**	$5.91E^{-07}$	0.000431662	2.907037
**trabecula carnea hypoplasia**	$6.04E^{-07}$	0.000401312	3.418765
**impaired muscle contractility**	$1.00354E^{-06}$	0.0005643	2.080982
**thin ventricular wall**	$1.1203E^{-06}$	0.000584957	2.649932
embryonic growth arrest	$1.38036E^{-06}$	0.000593557	2.093131
**heart left ventricle hypertrophy**	$1.5073E^{-06}$	0.000612129	3.751783
**ventricle myocardium hypoplasia**	$1.63216E^{-06}$	0.000596556	2.886228
**abnormal ventricle myocardium morphology**	$2.56739E^{-06}$	0.000893695	2.501817
**increased heart ventricle size**	$2.8742E^{-06}$	0.000955019	2.375723
**abnormal endocardium morphology**	$3.38901E^{-06}$	0.001077116	4.093677
**cardiac hypertrophy**	$5.88302E^{-06}$	0.001654033	2.365554
**abnormal myocardial fiber morphology**	$1.61626E^{-05}$	0.003692146	2.163135
disorganized extraembryonic tissue	$1.88935E^{-05}$	0.00394604	6.282186
abnormal placenta labyrinth morphology	0.000045231	0.008064356	2.227896
**abnormal myocardial trabeculae morphology**	$4.52477E^{-05}$	0.007875247	2.164085
embryonic lethality before turning of embryo	0.000137408	0.017318121	2.470141
spleen hypoplasia	0.000144694	0.017339512	2.303841
**abnormal fetal atrioventricular canal morphology**	0.000218274	0.023124369	2.127119
abnormal liver sinusoid morphology	0.000302493	0.026641207	3.200358
increased fetal size	0.000320088	0.027207472	5.584437
**abnormal atrioventricular cushion morphology**	0.000583518	0.043085989	2.092604
increased spleen weight	0.000682178	0.04660485	2.351304
abnormal hypersensitivity reaction	0.000733219	0.048286771	2.225326
abnormal hepatobiliary system development	0.00077956	0.04955292	2.266412

Supplementary Table 4. Gene markers used in Quadrant analysis	
**Gene Symbol**	**Quadrant Group**
Sca1/Ly6A	Cardiac Progenitor
Kit	Cardiac Progenitor
Kdr	Cardiac Progenitor
Abcg2	Cardiac Progenitor
Isl1	Cardiac Progenitor
Wt1	Cardiac Progenitor
CD31/PECAM1	Cardiac Progenitor
Flt1	Cardiac Progenitor
Abcb1a	Cardiac Progenitor
Tbx18	Cardiac Progenitor
Fut4	Cardiac Progenitor
CD34	Cardiac Progenitor
Acta2	De-Differentiation
Dstn	De-Differentiation
Runx1	De-Differentiation
Osm	De-Differentiation
Osmr	De-Differentiation
Msn	De-Differentiation
Myh7	Fetal gene response
Nppa	Fetal gene response
Nppb	Fetal gene response
Myh7b	Fetal gene response
Nfatc1	Fetal gene response
Adss1	Fetal gene response
Ppp3cb	Fetal gene response
Ccng1	Negative Regulator of Proliferation
Meis1	Negative Regulator of Proliferation
Gadd45A	Negative Regulator of Proliferation
Rb1	Negative Regulator of Proliferation
Mapk14	Negative Regulator of Proliferation
P38	Negative Regulator of Proliferation
Ccnd2	Proliferation
Fgf1	Proliferation
Cdk2	Proliferation
Cdkn3	Proliferation
Ccnd1	Proliferation
Ccna2	Proliferation

Gata4	Cardiac Transcription Factor
Gata6	Cardiac Transcription Factor
Mef2a	Cardiac Transcription Factor
Mef2c	Cardiac Transcription Factor
Tbx5	Cardiac Transcription Factor
Hand2	Cardiac Transcription Factor
Nkx2-5	Cardiac Transcription Factor

Supplementary Table 5. Sequences of primers used.

Primer Name	Sequence	Remarks	Expected size
Rplp0 Forward	actggtctaggacccgagaag	qPCR	124bp
Rplp0 Reverse	ctcccaccttgtctccagtc	qPCR	
LINCM3 Forward	ctcaagtgaaggcactgcaa	qPCR	125bp
LINCM3 Reverse	aggcacctcagaaacaaagg	qPCR	
LINCM9 Forward	aactttggggtgtcagtgatatg	qPCR	96bp
LINCM9 Reverse	cgttgcccaaagtaaatcca	qPCR	
Nppa Forward	cacagatctgatggatttcaaga	qPCR	68bp
Nppa Reverse	cctcatcttctaccggcatc	qPCR	
Dstn Forward	aggacgaaggtgcacagact	qPCR	132bp
Dstn Reverse	ttccgaactttcatgtcgtaga	qPCR	
Ccnd2 Forward	caccgacaactctgtgaagc	qPCR	71bp
Ccnd2 Reverse	tccacttcagcttacccaaca	qPCR	
Ccng1 Forward	tggacagattcttgtctaaaatgaag	qPCR	113bp
Ccng1 Reverse	cagtgggacattcctttcctc	qPCR	
LINCM1 RT PCR Fwd	gaggatggatggaacaggag	RT PCR	300bp
LINCM1 RT PCR Rev	gtcgcacggtcattgaact	RT PCR	
LINCM2 RT PCR Fwd	GCTGCATACAGAGAGGACTCAT	RT PCR	137bp
LINCM2 RT PCR Rev	GGTGTCTGCCACATCTTTGC	RT PCR	
LINCM3 RT PCR Fwd	acgtgttccatcctggtca	RT PCR	506bp
LINCM3 RT PCR Rev	ccagccaaatgaacaagca	RT PCR	
LINCM4 RT PCR Fwd	AGACAGTTGCTGTGGGTGTC	RT PCR	257bp
LINCM4 RT PCR Rev	GTCCACAAAACATTCTTTCCTTCTG	RT PCR	
LINCM9 RT PCR Fwd	gcaatctggacccaccagtt	RT PCR	165bp
LINCM9 RT PCR Rev	agatacgggaacggggagat	RT PCR	
LINCM6 RT PCR Fwd	CCAGCTGTCACTGGGTGTAT	RT PCR	145bp
LINCM6 RT PCR Rev	TACTGCGTGCAAGTGAGACA	RT PCR	
LINCM7 RT PCR Fwd	TAGAAACAGAACCGTGGGTTGA	RT PCR	102bp
LINCM7 RT PCR Rev	GGACTGTCCCTTCCTGAATTGT	RT PCR	
LINCM8 RT PCR Fwd	CTGAGTATTCGAGACCAACCTGT	RT PCR	563bp
LINCM8 RT PCR Rev	TTCGCAGATCTGTCTTGCAAAT	RT PCR	
LINCM5 RT PCR Fwd	AGGGTTCGATTCCGGAGAG	RT PCR	195bp
LINCM5 RT PCR Rev	ACCAGACTTGCCCTCCAATG	RT PCR	
LINCM10 RT PCR Fwd	AACTCTGGTGGAGGTCCGTA	RT PCR	287bp
LINCM10 RT PCR Rev	CACTAGGCACTCGCATTCCA	RT PCR	
LINCM11 RT PCR Fwd	CCCTCCGAAGTTTCCCTCA	RT PCR	92bp
LINCM11 RT PCR Rev	GGCCCCAAGACCTCTAATC	RT PCR	
LINCM12 RT PCR Fwd	CTCTCCACCTGTCTGCTTGC	RT PCR	599bp
LINCM12 RT PCR Rev	ATGGAAGGGGTGGGAAACA	RT PCR	
